# Perceived Emotional Synchrony in Collective Gatherings: Validation of a Short Scale and Proposition of an Integrative Measure

**DOI:** 10.3389/fpsyg.2020.01721

**Published:** 2020-07-31

**Authors:** Anna Wlodarczyk, Larraitz Zumeta, José Joaquin Pizarro, Pierre Bouchat, Fuad Hatibovic, Nekane Basabe, Bernard Rimé

**Affiliations:** ^1^Escuela de Psicología, Universidad Católica del Norte, Antofagasta, Chile; ^2^Faculty of Psychology, University of the Basque Country, Bilbao, Spain; ^3^Faculté de Psychologie et des Sciences de l’Éducation, Université catholique de Louvain, Louvain-la-Neuve, Belgium; ^4^Escuela de Psicología, Universidad de Valparaíso, Valparaíso, Chile

**Keywords:** perceived emotional synchrony, collective effervescence, collective gatherings, Durkheim, well-being

## Abstract

Over the past decade, there has been an increasing interest in the relationship between participation in collective gatherings and rituals and different important psychosocial variables and processes, such as social sharing of emotions, group cohesion, identity fusion, prosocial tendencies and behaviors, and well-being (e.g., Rimé, 2009; Xygalatas et al., 2013; Khan et al., 2015; Páez et al., 2015). These studies, coming from different lines of research, have proposed diverse explanatory mechanisms to explain the positive social and psychological effects of collective gatherings. In the present article, we focus on one of these mechanisms, known as collective effervescence, emotional communion, emotional entrainment, or perceived emotional synchrony (PES). First, we briefly discuss current conceptions of the emotional states and experience during collective gatherings and what they bring to the definition of PES. We close this point by proposing an integrative definition of PES. Second, structural validity of the original PES scale is examined. Third, incremental validity of PES is examined in two longitudinal studies, particularly with respect to well-being. Finally, we propose an integrative short form of the PES Scale, which measures antecedents and behavioral effects of collective effervescence.

## Introduction

### Perceived Emotional Synchrony and the Collective Gathering Experience

In this part, we examine different conceptions of the emotional experience lived by respondents during their participation in a group gathering that could help to elaborate our construct. Perceived emotional synchrony (PES) is a proxy for the notion of intense shared emotional experience introduced by Durkheim (1995) in the classic concept of collective effervescence. For Durkheim, collective effervescence is a shared or group state of high emotional arousal related to intensification of emotions by social sharing, felt in religious and secular collective rituals, irrespective of their content (joyful feasts or sad funerary rituals), which empowers the individual. Sociology of collective emotions also emphasizes that the experience felt during collective gatherings consists of a high mutually shared emotional arousal that emerges from all types of collective rituals; reinforces a sense of unison; and implies synchronization of emotional responses that, in turn, reinforces social cohesion (Collins, 2014; Von Scheve and Salmela, 2014). Von Scheve et al. (2017) evaluate the experience of participation in collective rituals using a scale of emotional entrainment referred to sport games (Football World Cup): (1) “How emotional have you felt about the…?” (2) “How much have you let yourself be carried away by the mood of other fans?” (3) “How much have you let yourself be carried away by the excitement of the World Cup?” In addition, questions about contextual factors facilitate a collective effervescence experience: (5) “How many of the games in which the … national team played did you watch at home with friends/family/acquaintances?” (6) “How many of the games in which the … national team played did you watch in a public pub/bar/restaurant?” (7) “How many of the games in which the … national team played did you watch at a large public viewing event or in the stadium?” Inspired by Collins’ model, sociologists measure the positive emotional experience of participation in religious rituals using questions about the participants’ positive (joy) and transcendent (awe, inspiration, and sense of God’s presence) emotions during a service (Draper, 2014). Social psychology approaches, related to social identity and self-categorization theory (SCT) also measure the effervescence experience of crowd and demonstrations using positive emotions (Hopkins et al., 2016). These authors emphasize the association of the experience felt by participants in a collective gathering with collective identity and the cognitive basis of enhanced social cohesion. SCT authors conceive of effervescence in crowds, demonstrations, and collective rituals as the extent to which participants judge their experience of participating in the collective gathering to be intense or extremely positive or felt intense positive emotions (Hopkins et al., 2016, p. 21). For instance, “My experiences in the crowd at the … demonstration have been emotionally intense” (Neville and Reicher, 2011). “In the period of pilgrimage, to what extent have you felt fulfilled, happy, alive and so on? (Hopkins et al., 2016) or experienced positive emotions ‘I felt joyful, I felt excited and I felt cheerful at … meeting”’ (Novelli et al., 2013). This conception is congruent with Durkheim’s ideas that collective gatherings can reinvigorate the individual. Indeed, because they are gathered together, group members communicate in the same thought and action; they feel a sense of comfort and enjoyment – for instance, ceremonies of mourning restore self-confidence, purpose of life, and well-being (Moscovici, 1988; Durkheim, 1995). In this approach, social identification is a contextual and process-based phenomenon conceived of as a sense of connection to a concrete set of co-present others or shared identity, grounded on a set of norms, values, and behaviors. Empirically, there is a positive and significant association between identity-related processes and effervescence or positive intense emotions in a ritual, demonstration, or meeting (Hopkins et al., 2016). Indeed, the emotional experience during collective gatherings is not only an intrapersonal individual emotional reaction, but also an interpersonal sharing of emotions (see Páez et al., 2007; Rimé, 2009). Nor is it only a consequence of cognitive categorization as group member. Indeed, Hopkins et al. (2016), although they describe identity-related processes as a precursor of the effervescence, recognize that an opposite path can actually occur; that is, conceptualizing shared identities resulting from emotional processes, such as PES, which could be an antecedent to the process of collective identification. Other psychologists also conceive the experience during collective gatherings as a positive emotional state but not limited to cognitive and social identification processes. Gabriel et al. (2017) posit that the collective effervescence experience occurs when a collective activity provides a feeling of connection to others in the crowd, a sense of engagement with something bigger than the self, and/or a “sensation of sacredness.” Their state measure of collective effervescence (Gabriel et al., 2017) includes three items appraising connections to others (e.g., “I felt connected to others who were present at the event”), one item appraising shared emotions (e.g., “I felt as if most everyone there felt the same emotions”). and four items for sensation of sacredness (e.g., “I felt as if the event changed me in some way”).

Other social psychologists observe that, in a collective gathering, emotional synchrony pulls humans fully but temporarily into the higher realm of the sacred, where the self disappears and collective interests predominate. The realm of the profane, in contrast, is the ordinary day-to-day world, where we live most of our lives, concerned about wealth, health, and reputation but nagged by the sense that there is, somewhere, something higher and nobler (Moscovici, 1988; Haidt, 2012). From our point of view, this sense of sacredness or the experience of being in contact with values and ideals that transform the person is a potential consequence of successful collective gatherings and probably is not an intrinsic component. Finally, the emotional state during collective gathering is also characterized by self-transcendent emotions. These emotions related to collectives (Haidt, 2012) include God’s presence, awe, inspiration or moral elevation, kama muta, and compassion (see Haidt, 2012; Draper, 2014; Fiske et al., 2017; Cusi et al., 2018; Pizarro et al., 2018). These emotional experiences are characterized by the decrease of self-absorption, the blurring of the barriers of the individual and the environment, the interpenetration of the individual self with the collective and greater connection with others and the world (Moscovici, 1988; Van Cappellen and Rimé, 2014). Table 1 summarizes features related to previously reviewed approaches; our conception relies on Durkheim’s original text.

**TABLE 1:** Summary of approaches to collective effervescence.

Authors	Durkheim	Sociology of emotions (von Scheve et al.)	Sociology of religion (Collins and Draper)	Social psychology (Social categorization theory)	Sacredness (Gabriel et al.)	Moscovici and Haidt
**Attributes**						
*• Group or Process State*	+	+				+
*• Shared*	+	+	+			+
*• High emotional arousal*	+	+	+	+		+
*• Irrespective of valence and form*	+					+
*• Unison/Connection*	+	+			+	
*• Positive emotions*			+	+		+
*• Transcendent emotions*			+			+
*• Connection with transcendent ideas and values*					+	+
**Key processes**	Collective effervescence/intensification and convergence of emotions	Emotional entrainment and Collective effervescence	Collective effervescence	Social identification and categorization	Connection with sacredness	Homo duplex, connection with values and something bigger than the self

### Conditions of Emergence

The emotional experience during collective gatherings is influenced by a wide variety of variables, some of them being indispensable criteria for it to occur (Collins, 2014). For instance, a necessary condition is the co-presence of other people physically gathered in a demarcated place and a degree of awareness of the presence and interaction with them (Collins, 2004). Higher social density or crowding probably reinforces and predicts PES (Liebst et al., 2019). Second, another antecedent is the focused and shared attention on one or more symbolic stimuli (Collins, 2014; Rennung and Göritz, 2016). A third antecedent is intentional coordination or behavioral synchrony among the participants in a given gathering. This coordination involves being in parallel (i.e., performing the exact same move) or interactive and complementary forms of interaction (Draper, 2014; Rennung and Göritz, 2016; Gabriel et al., 2017). These three conditions facilitate the emergence of a shared or common mood and emotions. As can be seen, all of these conditions are potentially met in a large set of collective rituals, such as in spiritual and religious pilgrimages (e.g., Tewari et al., 2012; Khan et al., 2015) or in marching rituals in the commemoration of a group historical events (e.g., Páez et al., 2015).

### Integrative Definition of Perceived Emotional Synchrony

Perceived emotional synchrony is a process that occurs when there is a collective gathering, shared focused attention, and behavioral synchrony that potentially elicits a collective emotional state. PES is a subjective process or the shared perception of entrainment, coordination, and synchronization of the affective experience. It is the emergence of high arousal emotions and, at least in part, of strongly positive emotions (joy, elation) in the participants in a successful collective gathering (Moscovici, 1988; Hopkins et al., 2016, 2019). PES, as a psychological experience, provides a feeling of connection to others participants and in-groups, a sense of engagement with something bigger than the self, and potentially a sensation of living in agreement with values and moral standards (Moscovici, 1988; Collins, 2004; Haidt, 2012; Gabriel et al., 2017)^1^.

### Measuring Perceived Emotional Synchrony

#### Reliability and Structural Validity of the PES Scale

Items appraise two main aspects of PES: emotional communion or intense sharing of emotions and feelings of unity (see Table 2). Exploratory and confirmatory factor analyses (CFA) found that a unidimensional structure fits well with the data using 16 items. The long version of the perceived emotional synchrony scale (PES-S), (Páez et al., 2015) is presented in Table 2 in both English and Spanish.

**TABLE 2:** Perceived emotional synchrony scale (PES-S) in English and Spanish.

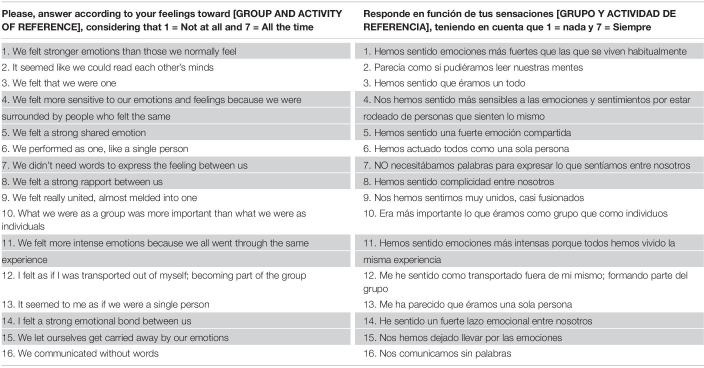

*Emotional communion: 1, 4, 5, 7, 8, 11, 14, and 15. Felt unity: 2, 3, 6, 9, 10, 12, 13, and 16.*

Factor analysis of PES has consistently resulted in a unidimensional structure. This structure has emerged in exploratory factor analyses of multiple samples, and CFA of the scale scores have resulted in adequate fit to a unique-factor model (Wlodarczyk et al., 2018).

## Study 1

### Psychometric Analysis of Measuring Perceived Emotional Synchrony

#### Method

##### Participants

We used a sample in the analysis of the structural validity of the PES. The group was Folk Festival-Tamborrada (Páez et al., 2015). A total of 550 volunteer participants (49.4% female) aged between 18 and 90 (*M* = 42.75 years, *SD* = 13.98), most of them (89.2%) residents of San Sebastian (Spain) and the rest of the people living around there, volunteered to complete the study forms. The sample was composed of several social groups, 57% married, 53% have children, 73% employed, and 11% unemployed.

##### Procedure

Town hall officials and coordinators of folkloric companies were contacted to recruit volunteers who would participate in the Tamborrada held on January 20, 2013. Longitudinal observations were analyzed in this study. Thus, encrypted personal e-mails were used to collect data online at three different measurement times (4 days before the celebration, the day of the celebration, and 4 days after).

##### Analysis

First, we computed reliability estimates, Cronbach’s alpha, and corrected item-total correlations using SPSS 21 and McDonald’s Omega using Omega, and we averaged these item-total correlations. We then created working scales containing the six items with the highest mean item-total correlations. A minimum alpha of 0.65 was desired; a previous work had referred to 0.65 alpha as satisfactory for a 5-item scale (see DeVellis, 1991).

#### Results

##### Reliability and descriptive statistics

Table 3 shows the univariate descriptive statistics of the original version of the PES scale composed of 16 items and adequate reliability indexes (both omega and alpha).

**TABLE 3:** Reliability estimates, descriptive statistics (*N* = 667).

Items	*M*	*SD*	95% CI		Variance	Skewness	Kurtosis (Zero centered)
			LL	UP			
Item 1	5.834	1.194	5.71	5.95	1.471	−1.252	1.867
Item 2	4.424	1.656	4.26	4.59	2.765	−0.347	–0.633
Item 3	5.316	1.488	5.17	5.46	2.252	−0.918	0.477
Item 4	5.614	1.336	5.48	5.75	1.827	−1.125	1.081
Item 5	5.811	1.219	5.69	5.93	1.533	−1.372	2.248
Item 6	5.189	1.519	5.04	5.34	2.342	−0.809	0.179
Item 7	5.308	1.522	5.16	5.46	2.351	−0.966	0.430
Item 8	5.753	1.168	5.64	5.87	1.411	−1.323	2.482
Item 9	5.347	1.387	5.21	5.49	1.960	−0.942	0.660
Item 10	5.680	1.343	5.54	5.81	1.846	−1.324	1.701
Item 11	5.669	1.267	5.54	5.80	1.650	−1.221	1.679
Item 12	4.912	1.623	4.75	5.07	2.661	−0.699	–0.216
Item 13	4.798	1.698	4.63	4.97	2.910	−0.557	–0.543
Item 14	5.307	1.469	5.16	5.45	2.192	−0.889	0.339
Item 15	5.193	1.526	5.04	5.35	2.359	−0.838	0.155
Item 16	4.979	1.576	4.82	5.14	2.512	−0.684	–0.220
McDonald’s Omega			0.977				
Cronbach’s alpha			0.967				

##### The structural validity of the PES

**FIGURE 1:**
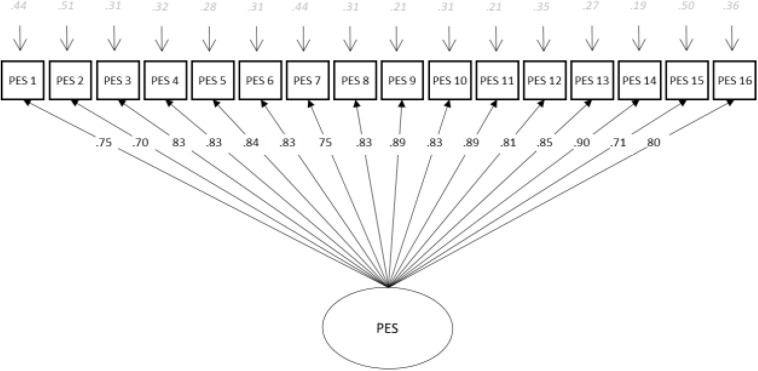
Confirmatory factor analysis of perceived emotional synchrony: 16 items.

Parallel analysis (PA) based on principal component analysis (Horn, 1965) with 500 random correlation matrices indicated that the advised number of dimensions is one. Additionally, the analysis of closeness to unidimensionality assessment (Ferrando and Lorenzo-Seva, 2018) revealed that the value of unidimensional congruence (UniCo) and item unidimensional congruence (I-UniCo) was 0.998 (BC BOOTSTRAP 95% CI = [0.997, 0.999]); scores larger than 0.95 suggest that data can be treated as essentially unidimensional. The value of explained common variance (ECV) and item explained common variance (I-ECV) was 0.954 (BC BOOTSTRAP 95% CI = [0.947, 0.970]), larger than the suggested value of 0.85 that confirms that the data can be treated as essentially unidimensional. Finally, the value of mean of item residual absolute loadings (MIREAL) and item residual absolute loadings (I-REAL) was 0.152 (BC BOOTSTRAP 95% CI [0.121, 0.166]), much lower than 0.300, which is the value that suggests that data can be treated as essentially unidimensional. Next, we assessed construct replicability by generalized H (g-h) index (Hancock and Mueller, 2001). High *H* values (>0.80) suggest a well-defined latent variable, which is more likely to be stable across studies. In our case, we obtained a highly H-latent value of 0.981 and H-observed of 0.932. H-latent assesses how well the factor can be identified by the continuous latent response variables that underlie the observed item scores, whereas H-observed assesses how well it can be identified from the observed item scores. Subsequently, driven by theory and previous analysis, we tested a unidimensional model of PES for both long and short versions by the mean of CFA (Muthén and Muthén, 1998-2012). In the first step, we tested the one-factor model as the model best representing the structure of PES. Next, we tested the same model, only with six items; the model showed good fit to the data as all three fit indices were close to the criteria (CFI above 0.95 and RMSEA close to 0.06) [Model fit: χ^2^ (100. *N* = 550) = 639.648, *p* < 0.001; CFI = 0.939; TLI = 0.927; RMSEA = 0.099 (95% CI [0.092, 106]); SRMR = 0.032]. The standardized estimates, i.e., factor loadings and factor correlation, are displayed in Figure 1.

**FIGURE 2:**
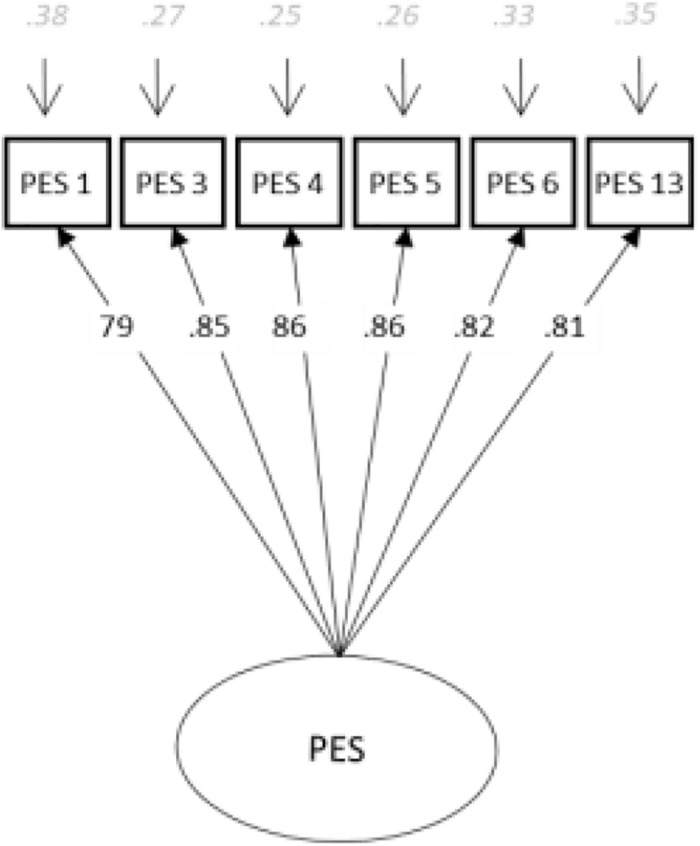
Confirmatory factor analysis of perceived emotional synchrony: 6 items.

Several considerations guided the construction of the short form. As in the development of other measures, we sought to maximize the reliability and validity of the instrument. Reliability and content validity, however, are often in conflict during the construction of short forms. Therefore, in addition to considering item-total correlations, our decisions regarding inclusion of items were also based on thorough examination of the content of individual items and within-scale factor analysis of the original scales. We included three items of felt unity (6, 13, and 3) and three of shared intense emotionality (4, 5, and 1) that had the highest coefficients with the latent variable. Item nine has a high load, but the semantic content overlaps with items of the scale of fusion identity (Gómez et al., 2011), and it was deleted to avoid confusion. Item 11 also has a high load, but the content includes two statements, and people can agree because of one or the other or both ideas, and answers are not clear. Finally, item 14 regarding felt unity also has a higher load, but content was redundant, and we chose items 6 and 13 that show better correlation with total items in the short version. The final model shows good fit indices [model fit: χ^2^ (100. *N* = 550) = 639.648, *p* < 0.001; CFI = 0.939; TLI = 0.927; RMSEA = 0.099 (95% CI [0.092, 0.106]); SRMR = 0.032]. The standardized estimates, i.e., factor loadings and factor correlation, are displayed in Figure 2.

## Study 2

### Incremental Validity

In addition to its reliability and structural validity, incremental validity of the PES scale was tested because the usefulness of any test is questioned if it cannot account for additional variance in relevant criteria such as well-being. When testing the incremental validity of PES, the primary concern was whether it was incrementally valid beyond theoretical constructs that explain the effects of collective gatherings, such as social identification, arousal, and rumination (Whitehouse and Lanman, 2014; Hopkins et al., 2016). Recently, psychosocial studies demonstrated that costly and relatively negatively toned collective rituals entail positive effects upon participants’ social (collective identity and social integration) as well as personal well-being. Many social rituals involve significant costs for participants, whether in the form of pain, physical effort, overcrowding, or living in difficult conditions, and they nevertheless engender positive psychosocial outcomes too. For instance, participants in a 1-month Hindu religious pilgrimage evidenced higher levels of collective identity and well-being compared to a control group (Tewari et al., 2012). How do costly rituals increase social cohesion and well-being? Whitehouse and Lanman (2014) propose that intense negative experiences are encoded as specific events in episodic memory. Such memories would favor rumination or “spontaneous exegetic reflection” on the significance of the unusual experience. Participants would, thus, develop webs of interpretation presumed to resemble co-participants’ thoughts and feelings, which would foster alignment and fusion of self and other (Whitehouse and Lanman, 2014). However, this high arousal and rumination inducing fusion of identity – and by this token, enhancing well-being – account that rests upon an intra-individual thinking process developed by each participant apart from co-participants contradicts Durkheim’s (1995) classic view that the cornerstone of social rituals lies in an inter-individual process of socially shared emotional experience or collective effervescence. In any case, PES should predict outcomes such as well-being above alternative explanatory variables such as social identification, arousal, rumination, and fusion of identity. These processes are analyzed in two relatively negative valence rituals: a patriotic parade aimed at enhancing negative emotions and aggressive dispositions toward national out-groups and a hazing ritual that enhances social identity evoking negative arousal.

### Study 2.1 Patriotic Parade May 21

This study was conducted around a patriotic paramilitary parade in Chile. On May 21, Iquique’s Naval Combat and the role of Arturo Prat are recalled through the organization of military civic parades between members of the Navy and students of various public and private schools. The school parades have pseudo-military characteristics because they imitate the instrumental and war bands of the Chilean armed forces. A territorial conflict was active in this year between Bolivia and Chile. A product of War of the Pacific (1879–1883), Bolivia was left without sovereign access to the sea, and an important part of its territory became part of Chile. Bolivians demanded a sovereign exit to the Pacific Ocean, and on the other side, Chile shows willingness to make commercial concessions but without territorial cession. Chile and Bolivia were in litigation in 2016 before the International Court of Justice of The Hague because Bolivia demands that the court force Chile to negotiate an exit to the sea. This patriotic parade is aimed to enhance nationalist and pro-war attitudes. This longitudinal study analyses if different mediators explain disposition to fight for the country and negative attitudes toward relevant national out-groups.

#### Participants and Procedure

Participants were secondary school students belonging to the municipal education of a working class neighborhood. Data collection took place in May 2016. The sample was composed of 65 students (44.2% women; aged between 15 and 18 years old; *M* = 16.86, *SD* = 0.61) from a high school in Puchuncaví (Valparaíso Region, Chile), who participated in the parade of the Naval Glories – also known as the 21st of May Parade. Participants completed self-reported measures at T1 (4 days prior the gathering), T2 (the day after participation), and T3 (4 days after the event). The students completed the questionnaires in the classrooms, in the computer room, and during the parade. The students did not gain any benefit from participating in the study.

#### Measures

We assessed two dependent variables that are supposed to be impacted by participation in collective rituals, i.e., participant’s negative emotions toward the out-group and disposition to fight for the nation in a war (Swann et al., 2009; Gómez et al., 2011).

Out-group negative emotions (based on Izard DES; Echevarría and Páez, 1989). Participants indicated the extent to which they feel negative anger and outrage toward Bolivians on a scale ranging from 1 (not at all) to 7 (very much). Correlations among the items were satisfactory (*r* = 0.757, *p* < 0.001 and *r* = 0.876, *p* < 0.001, pre- and post-measures).

Defense of Chile (adapted from Swann et al., 2009). Seven items measured the disposition to fight and sacrifice one’s life to protect Chile (“I’d do anything to protect Chile” or “I’d sacrifice my life if that saved the life of another Chilean”) on a 7-point scale (1 = totally disagree, 7 = totally agree). Reliability indexes were α = 0.902 and 0.949 for T1 and T3, respectively.

#### Mediators

Identity fusion with Chile (adapted from identity fusion – verbal scale, Gómez et al., 2011). Seven items were used to assess the fusion of identity of participants with Chile (e.g., “I am one with Chile”) in a 7-point scale (1 = totally disagree, 7 = totally agree). Reliability indexes were α = 0.875, 0.952, and 0.960 for T1, T2, and T3, respectively.

Chilean identity (adapted from in-group identification scale; Leach et al., 2008). Five items measured the national identification participants had (e.g., “I feel proud of being Chilean”) in a scale from 1 (totally disagree) to 7 (totally agree). Reliability indexes were α = 0.911 and 0.950 for T1 and T3, respectively. An indicator of changes in identification in the period of the parade was then computed (T3–T1).

Perceived emotional synchrony (short form of PES; Páez et al., 2015). We applied the final selection of six items to measure collective effervescence among participants to the collective gathering in T2 (α = 0.927).

#### Results

To assess the incremental validity of PES-S, we ran hierarchical linear regressions in three steps. First, we introduced a baseline measure of each dependent variable and the first mediator: changes in national identity between T1 and T3 (Step 1). In a second step, we introduced the second mediator: identity fusion of T2. Finally, in the third step, PES-S scores were included.

Results show that, for every analysis, baseline scores predict the criterion variables. In Step 2, when changes in Chilean identity and identity fusion was included, the last one explained every dependent variable – marginally in the case of negative emotions. Finally, at Step 3, the inclusion of PES-S was a significant predictor of defense of Chile, and fusion of identity was a significant predictor of negative emotions in the last step (see Table 4).

**TABLE 4:** Predictors of negative emotions and defense of Chile: hierarchical regressions.

		Negative emotions		Defense of Chile	
Step	Predictor	*b*	*p*	*b*	*p*
1	Variable at T1	0.639	0.0001	0.636	0.0001
	Changes in Chilean identity	−0.024	0.816	0.163	0.10
	Fit	*R*^2^ = 0.460***		*R*^2^ = 0.460***	
2	Variable at T1	0.593	0.000	0.446	0.0001
	Changes in Chilean identity	−0.078	0.462	0.104	0.281
	Identity fusion	0.197	0.076	0.345	0.004
	fit	*R*^2^ = 0.405***	Δ*R*^2^ = 0.034	*R*^2^ = 0.533***	Δ*R*^2^ = 0.099**
3	Variable at T1	0.590	0.0001	0.558	0.0001
	Changes in Chilean identity	−0.079	0.456	0.097	0.282
	Identity fusion	0.279	0.050	0.059	0.684
	PES-s	−0.126	0.345	0.351	0.004
	Fit	*R*^2^ = 0.407***	Δ*R*^2^ = 0.01	*R*^2^ = 0.592***	Δ*R*^2^ = 0.065**

**p < 0.01; ***p < 0.001.

#### Study 2.2 Students’ Hazing

This study was conducted on the campus of the University of Louvain, addressing newcomer hazing, a costly ritual (physical efforts, disgusting stimuli, humiliations…) frequently practiced in (a.o.) fraternities, sororities, military organizations, or athletic groups. To illustrate, participants may have to crawl through a muddy field while someone curses and yells at them. Such practices are worrisome due to their potential danger, whereas advocates stress social cohesion outcomes (e.g., Campo et al., 2003). Depending on the fraternity, hazing lasts from 2 to 4 weeks.

#### Participants

One hundred twenty freshmen (*M*_age_ = 18.74, *SD* = 1.00, with an equal number of men and women) linked to different hazing committees, depending on their area of origin and type of study, participated in all three measurement times: (1) baseline levels of dependent variables assessed just before hazing, (2) potential mediators halfway through hazing, (3) dependent variables 3 days after the final hazing ceremony. Questionnaires were paired using a specific code for each participant.

#### Measures

We assessed variables that were found to be impacted by participation in a collective ritual: participant’s identity fusion with the other hazed students (Swann et al., 2009) and individual and social well-being–related variables, psychological well-being (Vázquez and Hervás, 2012), and positive emotional climate (De Rivera and Páez, 2007). Unless specified, all items were rated on 5-point scales ranging from 1 (=“not at all”) to 5 (=“very strongly”). Completing the online questionnaires took, on average, 7 min for the questionnaire at time (1), 5 min for the questionnaire at time (2), and 7 min for the questionnaire at time (3). Main dependent variables were measured at times (1) and (3). Potential mediators were evaluated at time (2).

Identity fusion (Swann et al., 2009). A second indicator of social cohesion used a pictographic measure appraising “identity fusion” with the other participants. Five pictures showed different degrees of overlap between a smaller and a larger circle representing, respectively, “the self” and “the group” (1 = no overlap, 5 = complete overlap). Participants chose the diagram “that best describes the relationship between you and the other rookies.”

Well-being (a short form of the Pemberton happiness scale; Vázquez and Hervás, 2012). A six-item version was used (e.g., “I feel very connected to the people around me,” “I think my life is useful and valuable,” “I enjoy the little things of everyday life”), yielding a single indicator of well-being, α = 0.76 at time (1) and 0.81 at time (3).

Emotional climate (Páez et al., 2007). We used two items to measure participants’ perceptions of the main shared emotions in their social milieu in terms of positive trust among participants and solidarity (1 = totally disagree; 5 = totally agree). Reliability was *r* = 0.426, *p* < 0.001 and *r* = 0.330, *p* < 0.001 (for positive climate at T1 and T3).

#### Mediators

The first mediator is a measure of change of social identification during the hazing. The second and third mediators, experience-elicited arousal and experience-related thoughts, constitute proxy measures for the high-arousal intrapersonal model of costly ritual. The fourth one, PES, aims at appraising the model based on the socially shared experience.

Social identification (a short version of the in-group identification scale, Leach et al., 2008) was measured using six items assessing solidarity (“I feel solidarity with my co-rookies,” “I feel committed to my co-rookies”), satisfaction (“I am glad to be a rookie,” “It is pleasant to be a rookie”), and centrality (“I often think about the fact that I am a rookie,” “The fact that I am a rookie is an important part of my identity”): α = 0.47 and 0.76 for T1 and T3, respectively^2^. An indicator of the change in identification in the period of hazing was then computed (T3–T1).

Experience-elicited arousal (*ad hoc*). Three questions were used: “Overall, how much stress or trauma did you experience during the hazing?” “How intense was this experience for you?” “To what extent did you feel anxiety during this experience?” Ratings (1 = very weak; 5 = very strong) were averaged in a single index, α = 0.72.

Experience-related thoughts (*ad hoc*). Three questions assessed respondents’ thinking of the ritual (0 = not at all, 4 = very much). “Since the event, to what extent has it occupied your mind?” “Do you ever experience thoughts, mental images, memories about hazing?” “To what extent do these thoughts monopolize your attention?” An average thought score was established, α = 0.70.

Perceived emotional synchrony (PES-S, a short form of PES; Páez et al., 2015). Six items extracted from the 18-item scale assessed the experience of emotional effervescence. An average score of PES was computed, α = 0.88.

#### Results

To assess the incremental validity of PES, we ran hierarchical linear regressions as in the previous study, but in a second step, we introduced as mediators experience-elicited arousal and experience-related thoughts. In the third step, PES was introduced. Results showed that PES was always significantly linked to the level of the dependent variable at time (3) with a significant *R*^2^ change ranging from 0.018 to 0.036. By contrast, experience-elicited arousal and experience-related thoughts did reach significance levels only in the case of positive emotional climate (see Table 5).

**TABLE 5:** Predictors of identity fusion, well-being and social climate: hierarchical regressions.

		Dependent variables					
		Identity fusion IOS		Well-being		Positive emotional climate	
Step	Predictor	*b*	*p*	*b*	*p*	*b*	*p*
1	Variable at T1	0.459	0.000	0.649	0.000	0.625	0.000
	Shared Identity	0.378	0.000	0.418	0.000	0.259	0.000
	fit	*R*^2^ = 0.348***		*R*^2^ = 0.529***		*R*^2^ = 0.435***	
2	Variable at T1	0.458	0.000	0.653	0.000	0.596	0.000
	Shared Identity	0.378	0.000	0.424	0.000	0.264	0.000
	Arousal	0.120	0.169	−0.039	0.595	−0.137	0.098
	Rumination	−0.118	0.173	−0.056	0.453	0.050	0.540
	Fit	*R*^2^ = 0.351***	Δ*R*^2^ = 0.014	*R*^2^ = 0.528***	Δ*R*^2^ = 0.007	*R*^2^ = 0.439***	Δ*R*^2^ = 0.013
3	Variable at T1	0.409	0.000	0.614	0.000	0.443	0.000
	Shared Identity	0.365	0.000	0.409	0.000	0.254	0.000
	Arousal	0.103	0.224	−0.061	0.399	−0.164	0.048
	Rumination	−0.121	0.153	−0.055	0.447	0.034	0.674
	PES-s	0.197	0.010	0.173	0.008	0.145	0.048
	fit	*R*^2^ = 0.383***	Δ*R*^2^ = 0.036**	*R*^2^ = 0.553***	Δ*R*^2^ = 0.028**	*R*^2^ = 0.453***	Δ*R*^2^ = 0.018*

*b* represents standardized regression weights. * indicates *p* < 0.05; ** indicates *p* < 0.01; *** indicates *p* < 0.001.

## Discussion

Overall, the incremental validity of PES-S was confirmed through two studies on partially negatively valenced rituals, including a negative or costly ritual. Our studies emphasize the relevance of emotional synchronization in collective gatherings conducive to strong forms of social identification, particularly the overlapping of the individual with the collective self. However, studies also show the relevance of emotional sharing and of positive emotions.

### Integrative Scale for Collective Gatherings: Perceived Emotional Synchrony, Antecedents and Correlates

The short form includes three items of felt unity and three of shared intense emotionality. As antecedents, we included an item on shared focalized attention and Gabriel et al.’s (2017) question on behavioral synchrony. As a measure of a proximal potential outcome of PES (the experience that the event might help to transcend the ordinary) we used three of the original Gabriel et al. (2017) items. Item five was rewritten because, in a secular society, the concept of “sacred” is not easy to understand. In Gabriel et al.’s (2017) analysis, sacredness items did not fit well in a monofactorial structure, and we think that it is a complementary facet and a different construct of basic collective effervescence or PES (Table 6).

**TABLE 6:** An integrative measure of collective effervescence experiences.

“*Please, respond the following questions about the collective event in which you have participated”*
**Shared attention** (*ad hoc*) (based on Collins, 2014; Rennung and Göritz, 2016) (Ratings 1 = Nothing, 7 = A lot)
“The people in the collective gathering focused their attention on the same stimuli, symbols, objects or events (i.e., everyone focused simultaneously their attention or pay attention to the same aspect of the event at the same time)”
**Behavioral synchrony** (*ad hoc*) (based on Gabriel et al., 2017) (Ratings 1 = Nothing, 7 = A lot)
“The people in the event were involved in a synchronous activity at all (i.e., everyone doing the same thing at the same time, such as applauding, dancing, laughing, praying, cheering, or some other synchronous activity)”
**Perceived Emotional Synchrony – Short Version** (Páez et al., 2015). (Ratings 1 = Nothing, 7 = A lot) *During the event, to what extent have you felt:*
1. We performed as one, like a single person
2. It seemed to me as if we were a single person
3. We felt that we were one
4. We felt more sensitive to emotions and feelings others that feel
5. We felt a strong shared emotion
6. We felt stronger emotions than those we normally feel
**Negative Emotional Arousal** (*ad hoc*) (Ratings 1 = very weak; 5 = very strong)
1. Overall, how much stress did you feel during this experience?
2. How intense was the collective experience for you?
3. How much anxiety did you feel during this experience?
**Intense Positive Emotions** collective gathering related (Novelli et al., 2013; Khan et al., 2015) (Ratings 1 = disagree strongly and 7 = agree strongly). *During the event, to what extent have you felt:*
1. Fulfilled
2. Happy
3. Alive
**Transcendent emotions** (Zickfeld et al., 2019 item 1, ratings 0 = not at all to 6 = a lot; Fredrickson, 2009 items 2 3 4 5, ratings 0 = not at all to 4 = extremely; and Páez et al., 1997 item 6 adapted from Positive emotional climate, ratings 1 = not at all to 6 = a lot) *During the event, to what extent have you felt:*
1. Moved, touched
2. Awe, wonder, or amazement in front of greatness
3. Morally inspired, uplifted, or elevated
4. Love, closeness, trust
5. Hopeful, optimistic, or encouraged
6. Feel solidarity
**Situated Social Identity** (Novelli et al., 2013) (Ratings 1 = disagree strongly and 7 = agree strongly) *Thinking in the group that perform the collective activity please answer the following statements*
1. I identified with the other members of the collective event
2. I am like the other people who were at the collective event
3. I felt strong ties with the other people who were at the collective event
**Identity Fusion** (Gómez et al., 2011) (Ratings 1 = totally disagree, 7 = totally agree) *Thinking in the group that performed the collective activity, please answer the following statements*
1. I’ll do for my group more than any of the other group members would do
2. I am strong because of my group
3. I make my group strong
**Transcendent Experience** (Gabriel et al., 2017) (Ratings 1 = disagree strongly and 7 = agree strongly)
1. I felt as if there was a greater purpose to the event
2. I felt as if there was something transcendent, associated to values and ideals, about the event
3. I felt as if there was something special about the event
4. I felt as if the event changed me in some way
**Rumination scale** (*ad hoc*) (Ratings 0 = not at all, 4 = very much)
1. Since the event, to what extent has it occupied your mind?
2. Do you ever experience thoughts, mental images, and memories about the event?
3. To what extent did these thoughts catch/grab/monopolize your attention?
**Social sharing in the aftermath of the event** (Rimé et al., 1991)
1. After the event, did you feel the need to talk about it with other people?
Not at all. □ 0 □ 1 □ 2 □ 3 □ 4 □ 5 □ 6 Extremely
2. How long after the event did you talk about it for the first time?
Within 2 h of the event The same day The next day Within 8 days Later on I haven’t talked about it until now
3. Since the event happened, how many times have you actually talked about it?																				
Never 1–2 times 3–4 times 5–6 times 7–8 times 8–10 times 10–20 times More than 20 times	
4. Since the event happened, how many different people have you talked with about it?																				
None 1 2 3 4 5 6 or more
5 Currently, do you feel like talking about this event?																				
Not at all. □ 0 □ 1 □ 2 □ 3 □ 4 □ 5 □ 6 Extremely	

*Antecedents: shared focalized attention and behavioral synchrony. Correlates as proximal potential outcome of perceived emotional synchrony: Intense positive emotions, Transcendent emotions, Negative Emotional arousal, Correlates as Outcomes: identity and beliefs (Situated social identification, Fusion of identity with others, and Transcendent Experience). Interpersonal Correlates: Rumination, a scale of repetitive thoughts, Social sharing after the event.*

Another element that is emphasized in the experience of collective gatherings and demonstrations is the joy of being together or the positive valence of being together (Durkheim, 1912/2008; Moscovici, 1988; Hopkins and Reicher, 2016). We propose three items used in the study of concerts, demonstrations, and collective religious rituals (Novelli et al., 2013; Khan et al., 2015). Some authors argue that participation in collective gatherings and the experience of collective effervescence or PES are intrinsically related to self-transcendent emotions such as kama muta or being moved by love to/for others (Fiske et al., 2017) or social awe and moral inspiration (Haidt, 2006). To tap into these proximal emotional effects, we use items of the emotional positivity scale (Fredrickson, 2009), one item of the Kammus scale (Fiske et al., 2017) and one item of positive emotional climate (Páez et al., 1997).

To assess the process of social identification with other participants in the collective gathering, we add three items used by SCT authors (Novelli et al., 2010), and for the process of fusion of identity with others participants, three items of the Gómez et al. (2011) verbal scale that did not overlap semantically with PES and social identification. Because some authors, such as Xygalatas et al. (2013), emphasize negative emotional arousal as an important explanatory variable of collective rituals, at least for costly and negative valence rituals, and because some positive valence rituals imply a challenge and effort, we include a measure of negative arousal (Bouchat et al., 2020). In the same vein, because the intrapersonal cognitive process or rumination, together with arousal, are supposed to be explanatory variables of collective gathering effects, a scale of repetitive thoughts was included (Whitehouse and Lanman, 2014). Finally, social sharing of emotions and capitalization play an important role in the process of well-being, post stress growth, construction of emotional climate, and collective memories (Rimé, 2007), a measure of frequency of social sharing was included.

## Conclusion

How do instances of collective gatherings as collective rituals, secular, and religious ceremonies, enhance well-being even in the case of a negatively valenced event? We propose that collective effervescence or PES is one of the main explanatory variables of this effect. PES is a process that occurs when there is a collective gathering, shared focused attention, and behavioral and emotional synchrony. It is the shared perception of entrainment, coordination, and synchronization of the affective experience. This group state of high emotional arousal related to intensification of emotions, irrespective of the content and form of the collective gathering, evokes unison and connection with others and empowers the individual.

A short form of PES was described in this paper. Reliability and content validity, however, are often in conflict during the construction of short forms. When questionnaire items are chosen for inclusion in a short form based solely on high item-total correlations, the result is often a scale that measures only a narrow portion of the original construct, a phenomenon referred to as the “attenuation paradox” (Loevinger, 1954). In the short form, we opted for avoiding overlapping with fusion of identity and social identification as well as for including inclusive items of shared intense emotions and felt unity. The scale shows satisfactory structural validity and incremental validity in two longitudinal studies.

Contact with sacred values and transcendent emotions or a collective identity process, like social identification or cognitive categorization as in-group members, or a more affectively loaded process of collective identity such as fusion of identity, were excluded as items and contents because they are potential outcomes or alternative explanatory variables.

Our results clearly favor the inter-individual process of socially shared emotional experience over the intra-individual cognitive process such as social identification and rumination even regarding effects of relatively negatively valenced and costly rituals. More specifically, we showed that PES always significantly increases the explained variance of all but one dependent variable above and beyond other factors (i.e., social identification, identity fusion, arousal, rumination).

When people are gathered together in a costly ritual, an out-group anger-related parade or a funerary ritual, group members communicate in the same thought and action, they share and synchronize emotion. A successfully conducted collective gathering that induces a middle to high level of PES helps in the creation of an emotional atmosphere or climate, which is the expression of how the majority of people feel regarding the group’s current situation (De Rivera and Páez, 2007) as was found in the two longitudinal studies in which PES predicts positive emotional climate. This intensification and convergence of emotions fueling a sense of comfort and enjoyment restores self-confidence, purpose of life, and well-being (Moscovici, 1988; Durkheim, 1995), and in fact, PES predicts psychological well-being in the study. In other terms, collective effervescence measure by our PES scale predicts a higher level of positive collective emotions and of personal or psychological well-being, this means collective and individual well-being.

Far from excluding other variables, our results suggest that PES adds some explanatory power to a multifactor mechanism. For instance, social identification is probably a necessary condition for fusion, and both processes are relevant as shown in other studies. In fact, the baseline level of social identification shows positive correlations with PES (*r* = 0.49 and *r* = 0.44 in section “STUDY 1” and section “STUDY 2,” respectively, and with fusion of identity *r* = 0.62 in section “STUDY 2”). Social identity, as an internalized sense of shared group membership, and an associated sense that one is part of a bigger “us” is related to low depression and well-being (Steffens et al., 2017; Postmes et al., 2018).

Fusion of identity plays a specific role by reinforcing negative emotions toward out-groups, confirming the affective load facet of this process (Gómez et al., 2011). On the other hand, some evidence supports the role of negative arousal as a mechanism explaining the positive outcomes of some type of rituals. In our view, PES explains a complementary part of the variance and suggests that a neo-Durkheimian model of the positive effects of participation in collective gatherings is a valid one. These results also do not dispel intrapersonal experiences of high-arousal rituals. That emotional experiences elicit recurrent thoughts is well documented (e.g., Rimé, 2007, 2009) as well as that negative emotional arousal during collective rituals such as demonstrations predicts positive outcomes such as posttraumatic collective growth at the bivariate level (Rimé, 2009). For instance, participants in Hindu religious rituals involving body piercing evidenced higher levels of prosocial attitudes and inclusive social identity, and these effects were proportional to the intensity of their reported suffering (Xygalatas et al., 2013).

Nevertheless, in the present data, an interpersonal and group process like PES demonstrates its superiority in predicting the effects even of costly rituals. Our studies are not alone in reaching this conclusion. Lobato and Sainz (2019) observed participants’ identity fusion after a pilgrimage, thus further confirming that costly rituals entail social cohesion. Long-term effects of social cohesion were not predicted by intrapersonal variables (material remembrances; symbolic memories; reminiscent thoughts) but well by contacts with other pilgrims. Even in costly rituals and other negatively valenced rituals, positive shared emotions could fuel well-being (Tewari et al., 2012). What matters is the creation of a positive emotional atmosphere in which grief, sadness, anger, and fear are transformed into hope, solidarity, and trust (Durkheim, 1995; Collins, 2004). In sum, rituals reinforce emotions, particularly positive collective emotions, such as awe, moral inspiration, and hope, through PES, and this is how they strengthen social cohesion and increase well-being (Durkheim, 1995; Collins, 2004). The collective rituals have social consequences in the relations between nations and social groups, national identities need patriotic rituals to share a sense of pertinence and sacred values and to perpetuate intergroup conflicts (as is shown in this Chilean ritual study). Instead, rituals celebrating global identity promote social solidarity and peace attitudes (De Rivera and Carson, 2015). In sum, collective rituals, irrespective of their emotional content (e.g., joyful feasts, sad funerary, or patriotic rituals) empower the individual, reinforce social ties, and motivate moral commitment to groups, to leaders, and to values (Rai and Fiske, 2011; Páez et al., 2015; Cusi et al., 2018; Pizarro et al., 2018).

In conclusion, our review and empirical studies emphasize the relevance of emotional synchronization in collective gatherings conducive to strong forms of social identification, particularly the overlapping of the individual with the collective self. However, studies also show the relevance of emotional sharing and of positive emotions. Globally, our review of models and studies reaffirm the importance of shared emotional experiences during collective gatherings and rituals, justifying the evaluation and measurement of the construct of perceived emotion synchrony as a proxy indicator of these processes of collective effervescence.

## Data Availability Statement

The raw data supporting the conclusions of this article will be made available by the authors, without undue reservation.

## Ethics Statement

The studies involving human participants were reviewed and approved by Ethical Committee of the University of the Basque Country. The patients/participants provided their written informed consent to participate in this study.

## Author Contributions

AW, LZ, PB, JP, and FH planned and collected the data. NB and BR were involved in planning and supervised the work. All authors processed the data, performed the analysis, drafted the manuscript, designed the figures, performed the calculations, discussed the results, and commented on the manuscript.

## Conflict of Interest

The authors declare that the research was conducted in the absence of any commercial or financial relationships that could be construed as a potential conflict of interest.

## Footnotes

1 This last facet and other features, such as intense positive and self-transcendent emotions, such as joy, sense of vitality, being moved by love, social awe, moral inspiration, enhanced collective identity and social cohesion, prosocial attitudes and behaviors, agreement with values and psychological well-being, we think are outcomes of PES more than facets of this phenomenon.

2 This low alpha likely results from the fact that, at the start, the status of “rookie” was totally new and alien to participants.
